# Eight Functional Polymorphisms in the Estrogen Receptor 1 Gene and Endometrial Cancer Risk: A Meta-Analysis

**DOI:** 10.1371/journal.pone.0060851

**Published:** 2013-04-08

**Authors:** Xin Zhou, Yang Gu, Ding-ning Wang, Sha Ni, Jun Yan

**Affiliations:** Department of Obstetrics and Gynecology, Shengjing Hospital of China Medical University, Liaoning, Shenyang, China; Baylor College of Medicine, United States of America

## Abstract

**Background and Objective:**

Emerging evidence indicates that common functional polymorphisms in the estrogen receptor 1 (ESR1) gene may have an impact on an individual’s susceptibility to endometrial cancer, but individually published results are inconclusive. The aim of this meta-analysis is to derive a more precise estimation of the associations between eight polymorphisms in the ESR1 gene and endometrial cancer risk.

**Methods:**

A literature search of PubMed, Embase, Web of Science and China Biology Medicine (CBM) databases was conducted on publications published before November 1^st^, 2012. Crude odds ratios (ORs) with 95% confidence intervals (CIs) were calculated. Statistical analyses were performed using the STATA 12.0 software.

**Results:**

Thirteen case-control studies were included with a total of 7,649 endometrial cancer cases and 16,855 healthy controls. When all the eligible studies were pooled into the meta-analysis, the results indicated that PvuII (C>T) polymorphism was associated with an increased risk of endometrial cancer, especially among Caucasian populations. There were also significant associations between rs3020314 (C>T) polymorphism and an increased risk of endometrial cancer. Furthermore, rs2234670 (S/L) polymorphism may decrease the risk of endometrial cancer. However, no statistically significant associations were found in XbaI (A>G), Codon 325 (C>G), Codon 243 (C>T), VNTR (S/L) and rs2046210 (G>A) polymorphisms.

**Conclusion:**

The current meta-analysis suggests that PvuII (C>T) and rs3020314 (C>T) polymorphisms may be risk factors for endometrial cancer, especially among Caucasian populations.

## Introduction

Endometrial cancer is the seventh most common cancer among women worldwide. An estimated of 287,100 women were diagnosed with endometrial cancer in 2011 [1]. Many studies have confirmed that genetic predisposition and environmental factors are involved in the etiology of endometrial cancer [2], [3]. However, the interaction between environmental factors and genetic susceptibility remains to be elucidated. Functionally relevant polymorphisms in genes involved in the sex hormone metabolic pathway may alter the exposure to exogenous sex hormones and affect the risks in endometrial cancer development [4]. To date, a number of single-nucleotide polymorphisms (SNPs) in sex hormone-related genes, including CYP11A1, CYP17A1, CYP19A1, CYP19, CYP1B1, UGT1A1, PGR, SHBG, AR, ESR1, etc, have been studied. Mutations in these candidate genes have already been linked to elevated risks in developing endometrial cancers [5]–[8].

The ESR1 gene encoding the estrogen receptor 1 is a newly identified oncogene for endometrial cancer [9]. The human ESR1 gene is located on chromosome 6, locus 6p25.1 and consists of approximately 300 kbps, including 8 exons and 7 introns [10]. Genetic and epigenetic changes in ESR1 gene may lead to differences in estrogen metabolism and thereby possibly explain inter-individual differences in endometrial cancer risk [11]. Therefore, it was hypothesized that polymorphisms in the ESR1 gene could be functional and were associated with endometrial cancer risk. A number of studies have been conducted to investigate the potential associations between common polymorphisms in ESR1 gene and endometrial cancer risk, such as rs2234693 (PvuII; C>T), rs9340799 (XbaI; A>G), rs3020314 (C>T), rs1801132 (Codon 325; C>G), rs4986934 (Codon 243; C>T), VNTR (S/L), rs2234670 (STR; S/L), and rs2046210 (G>A). The Pvu II polymorphism site is located on intron 1, 1400 bps upstream of exon 2, and the Xba I site is approximately 50 bps apart from the Pvu II site. The rs2234670 is also located on exon 1. The synonymous coding rs4986934 and rs1801132 SNPs are located on exon 3 and 4. The SNP rs2046210 is located 29 kb upstream from the first untranslated region (UTR) of the ESR1 gene. Most of the studies support the mechanism in which ESR1 gene mutations promote the development and progression of endometrial cancer by altering estrogen metabolism. However, there are also some studies suggesting that there exists no association between ESR1 gene mutations and their effects on susceptibility to endometrial cancer. A recent meta-analysis of 8 case-control studies by Wang et al have assessed the association between PvuII (rs2234693) and XbaI (rs9340799) polymorphisms of ESR1 gene and the risk of endometrial cancer. Their results indicate that PvuII polymorphisms may be associated with increased risk of endometrial cancer, especially among the Asian-Australian population [12]. However, the previous meta-analysis did not provide convincing and reliable evidences in associating ESR1 polymorphisms to endometrial cancer risk because it presented some obvious shortcomings. Firstly, some eligible studies were not searched and included in the previous meta-analysis, which resulted in their relatively small sample size. Secondly, only two polymorphisms (PvuII and XbaI) in ESR1 gene were evaluated in the previous meta-analysis, while the other common polymorphisms potentially related to endometrial cancer risk were not studied. Thirdly, the authors only performed subgroup analyses by geographical regions in exploring sources of heterogeneity in the previous meta-analysis. However, numerous other factors may also have caused the observed heterogeneity, such as differences in genotype methods, source of controls, ethnicity, etc. In view of the conflicting results from previous studies and the insufficient statistical power of the previous meta-analysis, we performed this updates meta-analysis to provide a more comprehensive and reliable conclusion by reevaluating the association between ESR1 gene polymorphisms and susceptibility to endometrial cancer.

## Materials and Methods

### Literature Search

Relevant papers published before November 1^st^, 2012 were identified through a search in Pubmed, Embase, Web of Science and China Biology Medicine (CBM) databases using the following terms: (“genetic polymorphism” or “polymorphism” or “SNP” or “single nucleotide polymorphism” or “gene mutation” or “genetic variants”) and (“endometrial neoplasms” or “endometrial neoplasm” or “endometrial cancer” or “endometrial carcinoma” or “endometrial tumor”) and (“estrogen receptor alpha” or “estradiol receptor alpha” or “estradiol receptor α” or “ER alpha” or “ERα” or “Estrogen Receptor 1” or “Estrogen Receptor α” or “ESR1” or “ESRα”). The references from the eligible articles or textbooks were also reviewed to find other potential sources. Disagreements were resolved through discussions between the authors.

### Inclusion and Exclusion Criteria

Studies included in our meta-analysis have to meet the following criteria: (a) case-control studies or cohort studies focused on the associations between ESR1 gene polymorphisms and endometrial cancer risk; (b) all patients diagnosed with endometrial cancer should be confirmed by pathological or histological examinations; (c) published data about the frequencies of alleles or genotypes must be sufficient. Studies were excluded when they were: (a) not a case-control study or a cohort study; (b) duplicates of previous publications; (c) based on incomplete data; (d) meta-analyses, letters, reviews or editorial articles. If more than one study by the same author using the same case series was published, either the studies with the largest sample size or the most recently published study was included. The supporting PRISMA checklist is available as supporting information; see Supplement S1.

### Data Extraction

Data from the published studies were extracted independently by two authors into a standardized form. For each study, the following characteristics and numbers were collected: the first author, year of publication, country, language, ethnicity, study design, numbers of subjects, source of cases and controls, pathological type, detecting sample, genotype method, allele and genotype frequencies, and evidence of Hardy-Weinberg equilibrium (HWE) in controls. In case of conflicting evaluations, disagreements were resolved through discussions between the authors.

### Quality Assessment of Included Studies

Two authors independently assessed the quality of included studies according to the modified STROBE quality score systems [13]. Forty assessment items related to quality appraisal were used in this meta-analysis with scores ranging from 0 to 40. Scores of 0–20, 20–30 and 30–40 were defined as low, moderate and high quality, respectively. Disagreements were also resolved through discussions between the authors. The supporting modified STROBE quality score system is available in Supplement S2.

### Statistical Analysis

Crude odds ratios (ORs) with 95% confidence intervals (CIs) were used to assess the strength of association under five genetic models: the allele model, the dominant model, the recessive model, the homozygous model, and the heterozygous model. The statistical significance of the pooled ORs were examined using the Z test. Between-study variations and heterogeneities were estimated using Cochran’s Q-statistic with a *P*-value <0.05 as statistically significant heterogeneity [14]. We also quantified the effect of heterogeneity by using the *I^2^* test (ranges from 0 to 100%), which represents the proportion of inter-study variability that can be contributed to heterogeneity rather than to chance [15]. When a significant Q-test with *P*<0.05 or *I^2^*>50% indicated that heterogeneity among studies existed, the random effects model (DerSimonian Laird method) was conducted for the meta-analysis. Otherwise, the fixed effects model (Mantel-Haenszel method) was used. To explore sources of heterogeneity, we performed subgroup analysis by cancer types, ethnicity, country, source of controls and genotype methods. We tested whether genotype frequencies of controls were in HWE using the *χ^2^* test. Sensitivity was performed through omitting each study in turn to assess the quality and consistency of the results. Begger’s funnel plots were used to detect publication biases. Egger’s linear regression test which measures funnel plot asymmetry using a natural logarithm scale of OR was also used to evaluate the publication biases [16]. All the *P* values were two-sided. All analyses were calculated using the STATA Version 12.0 software (Stata Corp, College Station, TX).

## Results

### Characteristics of Included Studies

**Figure 1:**
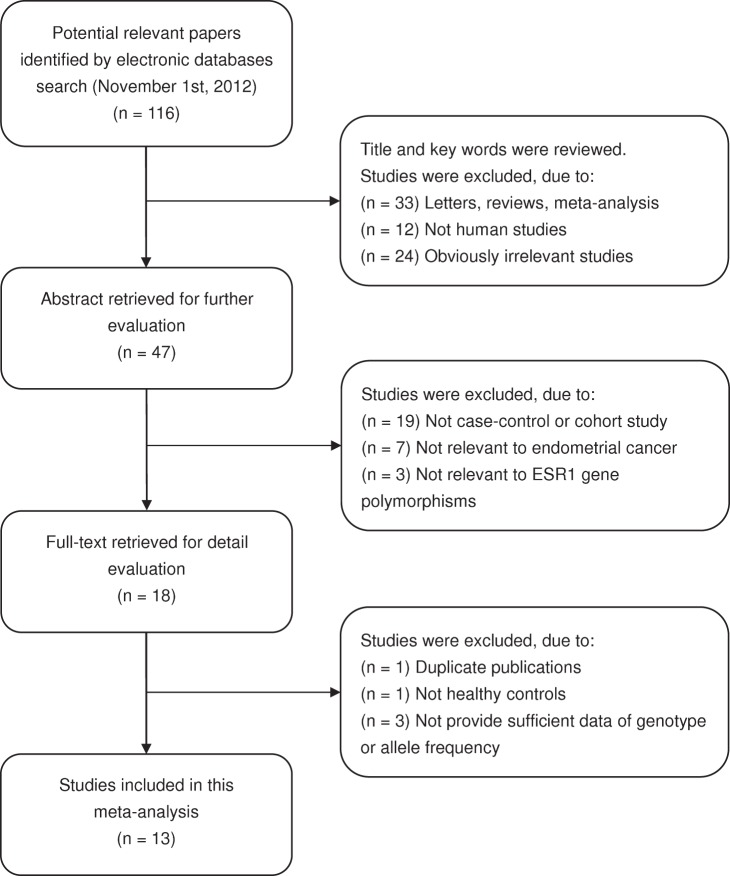
Flow chart of literature search and study selection.

In total, 116 articles relevant to the searched keywords were identified. Of these articles, 69 were excluded after reviewing their titles and key words; then, abstract and full text were reviewed, and another 34 papers were excluded. The details of the selection process were presented in a flow chart in Figure 1. Finally, thirteen case-control studies including 7,649 cases and 16,855 controls met the inclusion criteria in this meta-analysis [7], [8], [17]–[27]. The publication years of the involved studies ranged from 2000 to 2012. All patients diagnosed with endometrial cancer were also confirmed by pathological examinations. Six studies used hospital-based controls, while the other seven studies used population-based controls (community populations). Overall, five studies were conducted in Asian populations and eight studies in Caucasian populations. The DNA samples used for examination of ESR1 genetic polymorphisms were extracted from blood in ten studies and from tissues in the remaining three. Genotype methods include polymerase chain reaction-restriction fragment length polymorphism (PCR-RELP), direct DNA sequencing, BeadArray, TaqMan assay, and MassArray. Eight polymorphisms in the ESR1 gene were addressed, including rs2234693 (PvuII; C>T), rs9340799 (XbaI; A>G), rs3020314 (C>T), rs1801132 (Codon 325; C>G), rs4986934 (Codon 243; C>T), VNTR (S/L), rs2234670 (STR; S/L), and rs2046210 (G>A). HWE test was also conducted on the genotype distribution of the controls in all nine studies. Each study did not deviate from the HWE (all *P*>0.05). All quality scores of included studies were higher than 20 (moderate-high quality). The characteristics of the included studies are summarized in Table 1. The genotype distributions of eight polymorphisms in the ESR1 gene are presented in Supplement S3. Forest plot of ORs on the associations between all eight polymorphisms of ESR1 gene and endometrial cancer risk are showed in Supplement S4.

**Table 1:** Characteristics of included studies in this meta-analysis.

Firstauthor[Ref]	Year	Country	Ethnicity	Number		Source		Sample	Genotypemethods	Gene	SNP ID	Alternatenames	Qualityscores
				Case	Control	Case	Control						
Weiderpasset al [17]	2000	Sweden	Caucasian	261	380	PB	PB	Blood	PCR-RFLP	ESR1	rs2234693 (C)T)	PvuII	27
											rs9340799 (A>G)	XbaI	
											VNTR (S/L)	VNTR	
Sasakiet al [18]	2002	USA	Asian	113	200	HB	PB	Blood	DNA sequencing	ESR1	rs2234693 (C>T)	PvuII	
											rs4986934 (C>T)	Codon 243	
											rs1801132 (C>G)	Codon 325	
Iwamotoet al [19]	2003	Japan	Asian	92	65	HB	HB	Blood	PCR-RFLP	ESR1	rs2234693 (C>T)	PvuII	26
											rs9340799 (A>G)	XbaI	
Shanet al [20]	2003	China	Asian	52	52	HB	HB	Blood	PCR-RFLP	ESR1	rs9340799 (A>G)	XbaI	23
											rs2234693 (C>T)	PvuII	
Yanget al [21]	2007	China	Asian	87	90	HB	HB	Tissue	PCR-RFLP	ESR1	rs9340799 (A>G)	XbaI	24
											rs2234693 (C>T)	PvuII	
Wedrenet al [22]	2008	Sweden	Caucasian	702	1563	PB	PB	Tissue	DNA sequencing	ESR1	rs2234670 (S/L)	STR	26
											rs2234693 (C>T)	PvuII	
											rs9340799 (A>G)	XbaI	
											rs4986934 (C>T)	Codon 243	
											rs1801132 (C>G)	Codon 325	
Sobczuket al [23]	2009	Poland	Caucasian	120	120	HB	HB	Tissue	PCR-RFLP	ESR1	rs9340799 (A>G)	XbaI	24
											rs2234693 (C>T)	PvuII	
Ashtonet al [24]	2010	Australia	Caucasian	191	291	HB	PB	Blood	DNA sequencing	ESR1	VNTR (S/L)	VNTR	25
Sliwinskiet al [25]	2010	Poland	Caucasian	100	100	HB	HB	Blood	PCR-RFLP	ESR1	rs1801132 (C>G)	Codon 325	24
Yanget al [7]	2010	USA	Caucasian	417	407	PB	PB	Blood	BeadArray	ESR1	rs2234693 (C>T)	PvuII	32
											rs9340799 (A>G)	XbaI	
											rs4986934 (C>T)	Codon 243	
											rs1801132 (C>G)	Codon 325	
											rs3020314 (C>T)	–	
Li et al[27]	2011	China	Asian	935	947	PB	PB	Blood/Tissue	TaqMan assay	ESR1	rs2046210 (G>A)	–	34
Healeyet al [26]	2011	UK	Caucasian	4188	11928	HB	PB	Blood	MassArray	ESR1	rs3020314 (C>T)	–	33
Lundinet al [8]	2012	Sweden	Caucasian	391	712	HB	HB	Blood	TaqMan assay	ESR1	rs2234693 (C>T)	PvuII	31

Ref = reference; HB = hospital-based; PB = population-based; PCR-RELP = polymerase chain reaction-restriction fragment length polymorphism.

### Quantitative Data Synthesis

**Figure 2:**
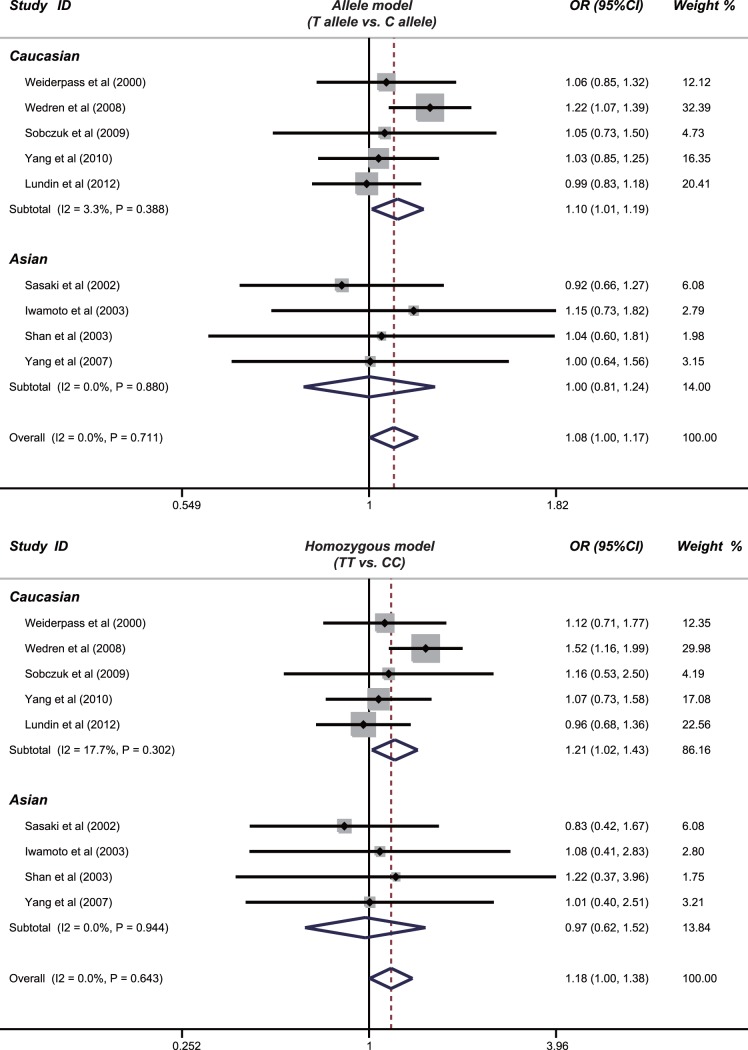
Forest plot of ORs by random effects model for ESR1 PvuII (C>T) polymorphism and endometrial cancer risk under the allele and homozygous models.

The association of PvuII (C>T) polymorphism with endometrial cancer risk is discussed in nine studies. The heterogeneity is not obvious (*P*>0.05), so the fixed effects model was used. The meta-analysis results showed that the PvuII polymorphism may increase the risk of endometrial cancer under the allele and homozygous models (T allele vs. C allele: OR = 1.08, 95%CI: 1.00–1.17, *P* = 0.043; TT vs. CC: OR = 1.18, 95%CI: 1.00−1.38, *P* = 0.043) (Figure 2). However, no associations were observed under the dominant, recessive and heterozygous models (all *P*>0.05). Further subgroup analyses showed that there were significant associations between PvuII polymorphism and endometrial cancer risk in Caucasian populations, population-based and DNA sequencing subgroups (as shown in Table 2).

**Table 2:** Meta-analysis of the associations between ESR1 PvuII and XbaI polymorphisms and endometrial cancer risk.

Subgroups	2 allele vs. 1 allele (allele model)			1/2+2/2 vs. 1/1 (dominant model)			2/2 vs. 1/1+ ½ (recessive model)			2/2 vs. 1/1 (homozygous model)			2/2 vs. ½ (heterozygous model)		
	OR	[95%CI]	*P*	OR	[95%CI]	*P*	OR	[95%CI]	*P*	OR	[95%CI]	*P*	OR	[95%CI]	*P*
rs2234693 (PvuII)															
Overall	1.08	[1.00, 1.17]	0.043	1.12	[0.98, 1.28]	0.098	1.11	[0.98, 1.25]	0.091	1.18	[1.01, 1.38]	0.043	1.08	[0.95, 1.23]	0.243
*Ethnicity*															
Caucasian	1.10	[1.01, 1.19]	0.030	1.12	[0.97, 1.30]	0.113	1.14	[1.00, 1.30]	0.047	1.21	[1.02, 1.43]	0.027	1.11	[0.97, 1.28]	0.129
Asian	1.00	[0.81, 1.24]	0.993	1.09	[0.77, 1.55]	0.615	0.92	[0.65, 1.30]	0.627	0.97	[0.62, 1.52]	0.902	0.88	[0.61, 1.28]	0.509
*Source of controls*															
Population-based	1.12	[1.02, 1.23]	0.023	1.20	[1.02, 1.42]	0.027	1.12	[0.97, 1.30]	0.116	1.26	[1.04, 1.53]	0.017	1.07	[0.92, 1.25]	0.400
Hospital-based	1.02	[0.89, 1.17]	0.792	0.97	[0.78, 1.22]	0.771	1.08	[0.87, 1.34]	0.489	1.01	[0.77, 1.34]	0.941	1.11	[0.88, 1.40]	0.379
*Genotype methods*															
PCR-RFLP	1.06	[0.91, 1.24]	0.473	1.13	[0.87, 1.48]	0.365	1.04	[0.81, 1.33]	0.767	1.12	[0.81, 1.54]	0.509	1.01	[0.77, 1.31]	0.968
DNA sequencing	1.17	[1.03, 1.32]	0.013	1.31	[1.06, 1.62]	0.014	1.18	[0.97, 1.42]	0.096	1.40	[1.09, 1.80]	0.009	1.09	[0.89, 1.33]	0.403
Other methods	1.01	[0.89, 1.15]	0.884	0.94	[0.75, 1.17]	0.557	1.09	[0.89, 1.33]	0.415	1.01	[0.78, 1.31]	0.950	1.12	[0.91, 1.39]	0.285
rs9340799 (XbaI)															
Overall	1.07	[0.89, 1.29]	0.459^†^	1.19	[0.97, 1.45]	0.099	1.07	[0.84, 1.36]	0.598^†^	1.15	[0.79, 1.67]	0.464^†^	1.11	[0.97, 1.27]	0.116
*Ethnicity*															
Caucasian	1.07	[0.89, 1.29]	0.462	1.16	[0.93, 1.43]	0.183	1.10	[0.86, 1.40]	0.451	1.13	[0.79, 1.63]	0.500	1.14	[0.99, 1.31]	0.079
Asian	1.06	[0.58, 1.93]	0.850	1.45	[0.79, 2.67]	0.232	0.96	[0.46, 2.00]	0.921	1.38	[0.35, 5.41]	0.642	0.94	[0.63, 1.42]	0.783
*Source of controls*															
Population-based	1.09	[0.88, 1.36]	0.435	1.17	[0.93, 1.48]	0.173	1.13	[0.87, 1.48]	0.368	1.17	[0.76, 1.81]	0.476	1.15	[0.99, 1.33]	0.055
Hospital-based	1.04	[0.70, 1.54]	0.848	1.23	[0.80, 1.88]	0.345	0.94	[0.56, 1.58]	0.828	1.15	[0.50, 2.65]	0.739	0.91	[0.64, 1.29]	0.600
*Genotype methods*															
PCR-RFLP	1.07	[0.82, 1.40]	0.607	1.17	[0.84, 1.62]	0.349	1.03	[0.72, 1.48]	0.861	1.15	[0.68, 1.95]	0.609	1.06	[0.84, 1.35]	0.625
DNA sequencing	1.26	[1.10, 1.46]	0.001	1.41	[1.03, 1.93]	0.033	1.33	[1.10, 1.60]	0.003	1.59	[1.14, 2.21]	0.006	1.28	[1.05, 1.55]	0.015
Other methods	0.88	[0.72, 1.09]	0.243	0.82	[0.51, 1.31]	0.402	0.87	[0.66, 1.14]	0.302	0.77	[0.48, 1.26]	0.303	0.89	[0.67, 1.18]	0.417

OR = odds ratios; 95%CI  = 95% confidence interval; 1 = wild allele; 2 = mutant allele; 1/1 = wild homozygote; 1/2 = heterozygote; 2/2 = mutant homozygote; *P_h_* = *P* value of heterogeneity test; † = estimates for random effects model.

Seven studies referred to the association between XbaI (A>G) polymorphism and endometrial cancer risk. The heterogeneity was significantly observed under the allele, recessive and homozygous models (*P*>0.05), which might result from the difference in ethnicity, country, source of controls and genotype methods, so the random effects model was used. Meta-analysis of these studies showed no significant associations between XbaI polymorphism and endometrial cancer risk under all five genetic models (all *P*>0.05). Stratified analyses also indicated no significant associations between XbaI polymorphism and endometrial cancer risk in each subgroup (as shown in Table 2). Although a significant association was found in DNA sequencing subgroup, this result might have lacked sufficient reliability due to the estimation error from the effect size of a single study.

Two of thirteen studies (involved eight clinical trials) reported results on the association between rs3020314 (C>T) polymorphism and endometrial cancer risk. All these two studies were conducted in Caucasian populations and based on community populations. When all the eligible trials were pooled into the meta-analysis, the results indicated that rs3020314 polymorphism might be associated with increased risk of endometrial cancer (T allele vs. C allele: OR = 1.05, 95%CI: 1.02−1.10, *P* = 0.007; TT+CT vs. CC: OR = 1.06, 95%CI: 1.01−1.11, *P* = 0.032; TT vs. CC+CT: OR = 1.10, 95%CI: 1.02−1.19, *P* = 0.020; TT vs. CC: OR = 1.12, 95%CI: 1.03−1.22, *P* = 0.007).

The Codon 325 (C>G) variation was investigated in four publications. Since heterogeneity was observed under the allele, recessive and heterozygous models (all *P*<0.05), the random effects model was used. The meta-analysis results showed that there were no significant associations between Codon 325 polymorphism and endometrial cancer risk under all five genetic models (G vs. C: OR = 0.82, 95%CI: 0.61−1.11, *P* = 0.195; GG+CG vs. CC: OR = 0.99, 95%CI: 0.85−1.15, *P* = 0.860; GG vs. CC: OR = 0.68, 95%CI: 0.38−1.24, *P* = 0.210; GG vs. CC: OR = 0.92, 95%CI: 0.66−1.27, *P* = 0.600; GG vs. CG: OR = 0.68, 95%CI: 0.37−1.26, *P* = 0.226). Subgroup analyses showed significant associations in hospital-based and PCR-RFLP subgroups (all *P*<0.05), but these estimates from a single study were also unreliable.

There were only three studies that referred to the associations between Codon 243 (C>T) polymorphism and endometrial cancer risk. All these studies were population-based, including two studies in Caucasian populations and one in Asian populations. We found no significant associations between Codon 243 (C>T) polymorphism and endometrial cancer risk under five genetic models (T allele vs. C allele: OR = 1.05, 95%CI: 0.80−1.36, *P* = 0.746; TT+CT vs. CC: OR = 1.05, 95%CI: 0.80−1.39, *P* = 0.715; TT vs. CC+CT: OR = 0.73, 95%CI: 0.16−3.37, *P* = 0.690; TT vs. CC: OR = 0.74, 95%CI: 0.16−3.39, *P* = 0.697; TT vs. CT: OR = 0.68, 95%CI: 0.14−3.18, *P* = 0.619). In the subgroup analysis by ethnicity, we also found no associations between Codon 243 polymorphism and endometrial cancer risk among both Caucasian and Asian populations (all *P*>0.05).

Furthermore, we have evaluated the associations of VNTR, STR (S/L) and rs2046210 (G>A) polymorphisms with endometrial cancer risk. There were only two studies referring to VNTR, and each one study referring to rs2234670 (S/L), and rs2046210 (G>A). Our results showed that rs2234670 (S/L) polymorphism may decrease the risk of endometrial cancer under the allele, recessive and homozygous models (L allele vs. S allele: OR = 0.87, 95%CI: 0.76−0.99, *P* = 0.040; LL vs. SS+SL: OR = 0.78, 95%CI: 0.62−0.99, *P* = 0.039; LL vs. SS: OR = 0.87, 95%CI: 0.76−0.98, *P* = 0.037), but these results were also extracted from a single study. However, there were also no significant associations of VNTR and rs2046210 (G>A) polymorphisms to the risk of endometrial cancer (all *P*>0.05).

### Sensitivity Analysis and Publication Bias

**Figure 3:**
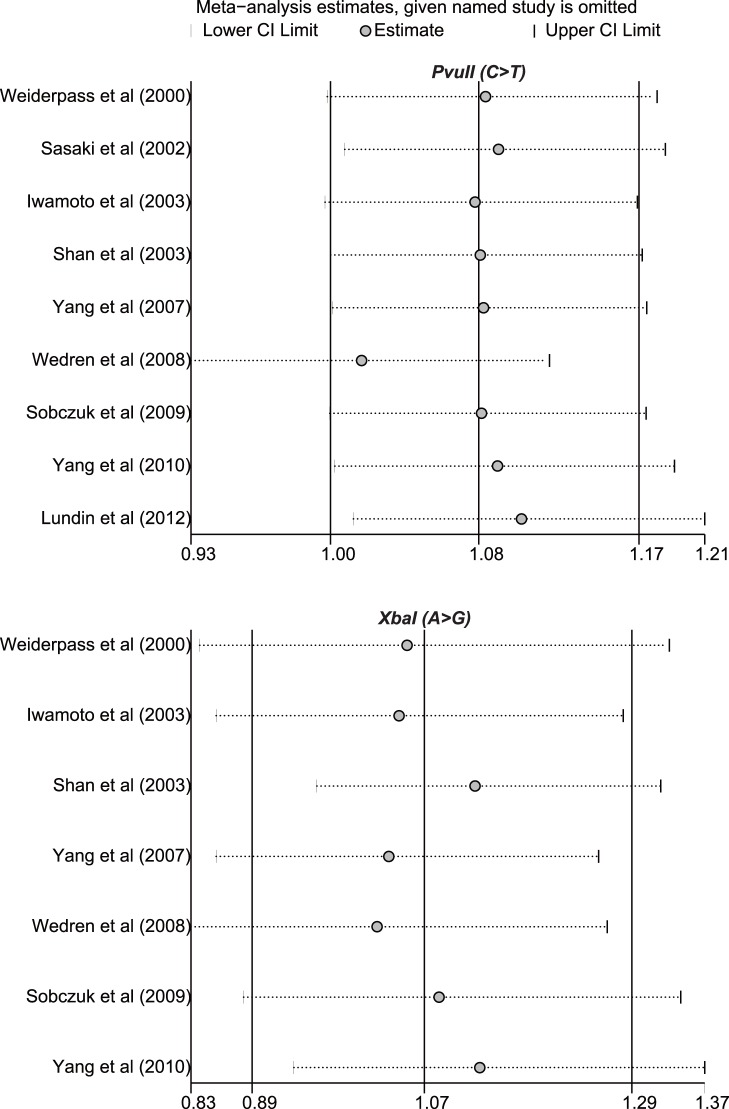
Sensitivity analysis of the summary ORs under the allele model on the association between ESR1 PvuII and XbaI polymorphisms with endometrial cancer risk. Results were computed by omitting each study in turn. Meta-analysis random-effects estimates (exponential form) were used. The two ends of the dotted lines represent the 95%CI.

Sensitivity analysis was performed to assess the influence of each individual study on the pooled ORs through omitting of individual studies. The analysis results suggested that no individual studies significantly affected the pooled ORs under any genetic models of PvuII (C>T) and XbaI (A>G) polymorphisms (Figure 3), indicating a statistically robust result.

**Figure 4:**
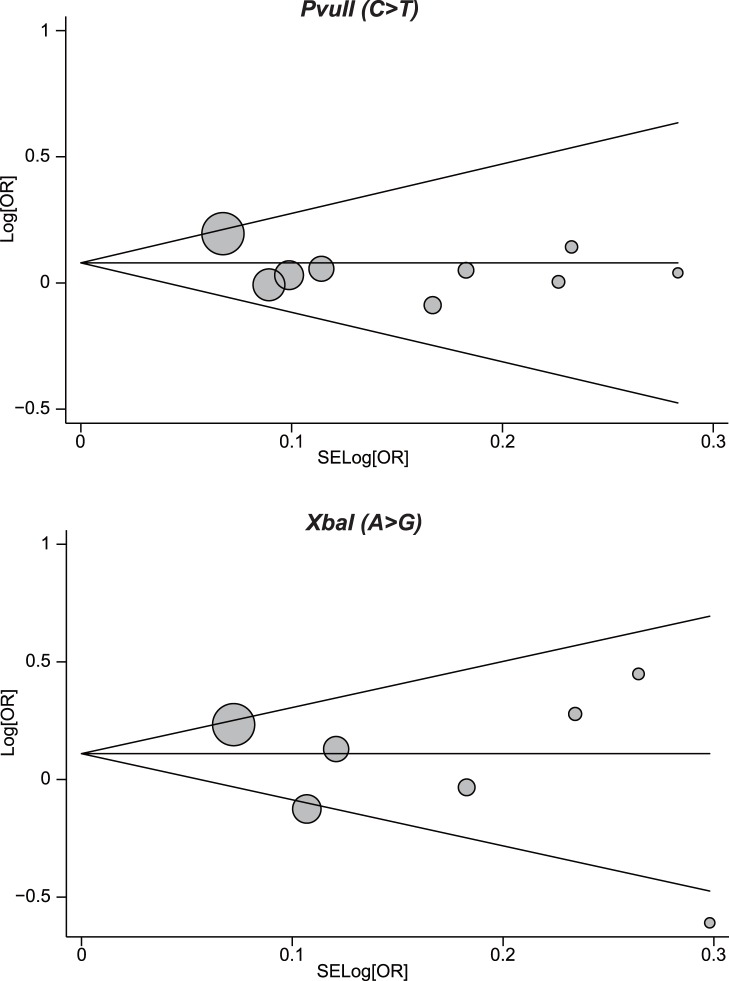
Begger’s funnel plot of the meta-analysis of ESR1 PvuII and XbaI polymorphisms and endometrial cancer risk. Each point represents a separate study for the indicated association. Log[OR], natural logarithm of OR. Horizontal line, mean magnitude of the effect. Note: Funnel plot with pseudo 95% confidence limits was used.

Publication biases within available research results might not be representative of all research results. Begger’s funnel plot and Egger’s linear regression test were performed to assess the publication biases of included studies. The shapes of the funnel plots did not reveal any evidence of obvious asymmetry under the dominant model of PvuII (C>T) and XbaI (A>G) polymorphisms (Figure 4). Egger’s test also showed that there was no strong statistical evidence of publication bias (PvuII: t = −1.31, *P*  = 0.232; XbaI: t = −0.73, *P*  = 0.496).

## Discussion

ESR1 gene is critical for hormone binding, DNA binding, and activation of transcription because it encodes an estrogen receptor that is key in the mediation of hormonal response in estrogen-sensitive tissues [24]. Thus, genetic mutations in ESR1 gene may contribute to its abnormal expression and are probably linked to increased risk of hormone-related cancers such as the breast, prostate, and endometrial cancers [28]. Li et al performed a meta-analysis that evaluated the association between ESR1 gene polymorphisms and breast cancer risk in diverse populations [29]. There results suggests that variant genotypes of PvuII and rs1801132 (Codon 325) may contribute to breast cancer susceptibility. Several ESR1 gene polymorphisms have been identified as candidates for prostate cancer susceptibility and among these, ESR1 PvuII and XbaI SNPs were suggested to possess significant associations with the development of prostate cancer [30]. Many previous genetic studies have suggested that ESR1 gene polymorphisms may play an important role in endometrial carcinogenesis [17]–[19], [22], [24], [25], [27], while other studies found no convincing evidence of these polymorphisms in increasing endometrial cancer susceptibility [7], [8], [21], [23], [26]. This controversy could be explained with several reasons, including the differences in study designs, sample size, ethnicity, statistical methods, etc. A recent meta-analysis by Wang et al indicated that PvuII polymorphism in the ESR1 gene could increase the risk of endometrial cancer, while XbaI polymorphism was not associated with susceptibility to endometrial cancer. However, they failed to assess the relationship between the other common polymorphisms in the ESR1 gene and endometrial cancer risk. This recent meta-analysis is aimed to update previous meta-analysis, as well as to provide a more comprehensive and reliable conclusion on the associations between eight common functional polymorphisms in the ESR1 gene and endometrial cancer susceptibility.

In this meta-analysis, 13 independent case-control studies were included with a total of 7,649 endometrial cancer cases and 16,855 healthy controls. When all the eligible studies were pooled into the meta-analysis, the results showed that PvuII (C>T) polymorphism was associated with increased risk of endometrial cancer, especially among Caucasian populations, while similar associations were not observed among Asian populations. Although the exact function of PvuII (C>T) polymorphism in the development of endometrial cancer among different populations is not yet clear, a possible reason could be that inherited mutations in ESR1 might be associated with changes in estrogen metabolism and thereby could possibly explain inter-individual differences in disease incidences of endometrial cancer [18]. However, no statistically significant associations were found between XbaI (A>G) polymorphism and endometrial cancer risk. The findings from this meta-analysis were consistent with the previous meta-analysis conducted by Wang et al, suggesting PvuII may be linked to the development of endometrial cancer. In addition to the previous meta-analysis, we also found a significant association between rs3020314 (C>T) polymorphism and an increased risk of endometrial cancer development, while the rs2234670 (S/L) polymorphism might decrease the risk of endometrial cancer development. Nevertheless, Codon 325 (C>G), Codon 243 (C>T), VNTR (S/L) and rs2046210 (G>A) polymorphisms showed no associations with the risk of endometrial cancer. These findings are consistent with the previous hypothesis that variability in the ESR1 gene may alter the risk of developing endometrial cancer, suggesting that they may be useful as biomarkers in predicting an individual’s genetic susceptibility to endometrial cancer.

Similar to other meta-analyses, our study also bears some limitations and shortages. First, the sample size is still relatively small and may not provide sufficient statistical power to estimate the correlations between ESR1 gene polymorphisms and endometrial cancer risk. Second, in this meta-analysis, potential sources of heterogeneity could include many other factors, such as age and sex structure, characteristics of healthy control, pre- and post-menopause, etc. Third, as a type of a retrospective study, a meta-analysis may encounter recall or selection bias, possibly influencing the reliability of our study results. Furthermore, in this meta-analysis, there is a significant difference in numbers between cancer cases and healthy controls, which may be one source of heterogeneity and may have some unfavorable effects on the reliability of our results. Finally, our lack of access to the original data from the studies limited further evaluation of potential interactions such as gene-environment and gene-gene interactions. In spite of these limitations, however, our present meta-analysis includes the largest number of eligible studies relevant to the relationship between ESR1 polymorphisms and endometrial cancer risk reported to date.

In conclusion, our meta-analysis suggests that PvuII (C>T) and rs3020314 (C>T) polymorphisms may be risk factors in endometrial cancer development, especially among Caucasian populations. These relationships will promise us a functional profiling of ESR1 gene and an understanding of the biological processes associated with endometrial cancer development and progression. It may also be further utilized as a diagnostic tool, as well as, an accurate determination of endocrine therapeutic strategies in endometrial cancer. However, further studies are still needed in order to validate the associations between polymorphisms in ESR1 and endometrial cancer.

## Supporting Information

Supplement S1 **PRISMA Checklist.**
(DOC)

Supplement S2 **The modified STROBE quality score systems.**
(DOC)

Supplement S3 **The genotype distributions of ESR1 polymorphism in case and control groups.**
(XLS)

Supplement S4 **Forest plot of ORs by random effects model for all eight polymorphisms of ESR1 gene and endometrial cancer risk under five genetic models: the allele model (A), the dominant model (B), the recessive model (C), the homozygous model (D), and the heterozygous model (E).**
(ZIP)
